# COVID-19-related stigma and its relationship with mental wellbeing: A cross-sectional analysis of a cohort study in Japan

**DOI:** 10.3389/fpubh.2022.1010720

**Published:** 2022-09-29

**Authors:** Emiko Sawaguchi, Sho Nakamura, Kaname Watanabe, Kanami Tsuno, Hiromi Ikegami, Naoko Shinmura, Yoshinobu Saito, Hiroto Narimatsu

**Affiliations:** ^1^Graduate School of Health Innovation, Kanagawa University of Human Services, Kawasaki, Japan; ^2^Cancer Prevention and Control Division, Kanagawa Cancer Center Research Institute, Yokohama, Japan; ^3^Department of Genetic Medicine, Kanagawa Cancer Center, Yokohama, Japan; ^4^Hygeia Communication General Incorporated Association, Kawasaki, Japan; ^5^Faculty of Sport Management, Nippon Sport Science University, Yokohama, Japan

**Keywords:** COVID-19, social stigma, emerging communicable diseases, quality of life, health communication, population health, vulnerable populations, risk factors

## Abstract

**Objective:**

Social stigma related to coronavirus disease (COVID-19), i. e., COVID-19 stigma, forms a burden on people socially, economically, and mentally. This study assessed COVID-19 stigma using a scale to identify a population likely to exhibit higher prejudice against COVID-19 itself as well as those infected with COVID-19.

**Methods:**

We adapted and modified the Cancer Stigma Scale to assess COVID-19 stigma and used it as the baseline survey of a cohort study in Japan. The questionnaire was disseminated to 1,573 participants (51.7% men) between December 2020 and March 2021. The questionnaire items included the infection status of individuals close to the respondent and their preventive behaviors related to COVID-19, quality of life (QOL; using the EuroQoL 5-Dimension 5-Level [EQ-5D-5L]), and psychological distress (using the 6-item Kessler Psychological Distress Scale [K6]). Exploratory and confirmatory factor analyses were performed to validate the COVID-19 stigma scale, and we further used the structural equation modeling (SEM) to assess the relationship with QOL and psychological distress.

**Results:**

COVID-19 stigma was calculated for the 257 (16.3%) participants who responded to the questionnaire. The mean age (standard deviation) was 54.5 (14.4) years, and 50.2% were men. Factor analysis revealed a five-factor model: Awkwardness (feeling uncomfortable being with a person infected before), Severity (fear of not being able to return to normal after infection), Avoidance (attitude of avoiding infected persons), Policy Opposition (expecting more public funding investment), and Personal Responsibility (believing that infected persons themselves are responsible for their infection). Participants > 70 years had the highest scores among other age groups considering all factors except for Policy Opposition. Standardized coefficients in SEM for COVID-19 stigma (latent variable) was highest for Severity (beta = 0.86). Regression coefficients of COVID-19 stigma on K6 and QOL were 0.21 (95% confidence interval [CI] 0.074–0.342) and −0.159 (95% CI −0.295–0.022), respectively.

**Conclusion:**

People aged ≥ 70 years are more likely to exhibit COVID-19 stigma. Additionally, the results indicate that COVID-19 stigma impacts QOL and psychological distress.

## Introduction

Coronavirus Disease (COVID-19) is an infectious disease caused by the severe acute respiratory syndrome coronavirus 2 that spread worldwide in 2020 and was declared a pandemic which is still ongoing (1, 2). As it was novel, no vaccine or evidence-based treatment had been established, and tremendous efforts were required to control and treat the infection. This led to a severe shortage of medical resources in many countries (3). Supplying a vaccine, specific treatment, or evidence-based treatment immediately after an outbreak is difficult, as was the case with past experiences of emerging infections such as the Ebola hemorrhagic fever (Ebola), severe acute respiratory syndrome, and Middle East Respiratory Syndrome. Consequently, an emerging infectious disease outbreak or pandemic induces fear and anxiety concerning infections (4–8). When this negative feeling about infections extends toward people who have been infected, those infected could be ostracized: negatively labeled, stereotyped, discriminated against, and persecuted (9–11). This phenomenon is called “social stigma.” Social stigma can be defined as prejudice or discrimination against patients, their families, or healthcare workers; it increases psychosocial burdens, leading to development of psychiatric symptoms such as anxiety and depression, thus decreasing the quality of life (QOL) (11, 12).

During the COVID-19 pandemic, social stigma associated with COVID-19 (COVID-19 stigma) was reported worldwide in early 2020 (5). Healthcare workers in 173 countries have experienced bullying due to COVID-19 stigma (13). In China, which experienced the earliest spread and global convergence of COVID-19, a positive association between COVID-19 stigma toward patients or their families and depressive symptoms and financial burden was reported (14). Research aiming to reduce COVID-19 stigma indicates that communication skills or keeping up with evidence-based information are essential in reducing stigma (15).

In a recent study from Japan, 23% of healthcare professionals reported experiencing COVID-19 stigma since January 2021 (16). In an effort to reduce COVID-19 stigma, public organizations and academic societies took measures such as issuing statements and introducing campaigns to honor healthcare professionals involved in patient care (17). The effectiveness of intervention measures to reduce COVID-19 stigma can be improved *via* a targeting or segmenting approach for the most vulnerable populations (18). For example, a study showed that providing educative content about the correct information regarding Ebola to younger populations through social networking services (SNS) resulted in successful spread of accurate information (19). The part of the population with a higher proportion of internet access showed lower infection rate, indicating that this intervention also contributed to the termination of infection (20). Thus, the target population must be identified and appropriate interventions provided to reduce social stigma, including COVID-19 stigma.

Identifying the target population involves targeting groups that are more likely to exhibit bias. Social stigma is measured for a wide range of diseases. A scale to measure social stigma was developed and validated for various diseases, including infectious diseases, psychological disorders, and cancer (21–23). By the end of March 2022, several studies had reported occurrence of COVID-19 stigma (5, 24, 25); however, no scale that could also measure associated stigma was validated for the Japanese population, and these studies did not focus on population groups more likely to exhibit bias against COVID-19 itself as well as people infected with COVID-19. In this study, we attempted to measure stigma associated with COVID-19 by applying an existing Japanese stigma scale for another disease. We used the Cancer Stigma Scale (CASS) for two reasons: First, the CASS was developed in a non-patient population as was the case for our study; second, since it includes items selected from a previous disease-related stigma scale incorporating Human Immunodeficiency Virus (HIV)/Acquired Immune Deficiency Syndrome (AIDS), leprosy, mental illness, epilepsy, and skin disease that were identified in a systematic review (21, 22). While the aforementioned disease-specific scale to assess social stigma exists, the reportedly assessed social stigma are similar among these scales, suggesting the need for a generic scale (21). The advantage of applying an existing scale for another disease is that, if successful, it can be potentially utilized for other emerging infectious diseases in the future, and would indicate the possibility of developing a generic scale.

Therefore, in this cross-sectional study, we developed and distributed a questionnaire survey on COVID-19 stigma during the COVID-19 pandemic to identify the population groups more likely to exhibit prejudice against COVID-19 itself and those infected with COVID-19. Furthermore, we tested our hypothesis that COVID-19 stigma increases psychological distress and decreases QOL in people infected with COVID-19, as indicated in other stigmas (11). Our findings may serve as evidence to show the benefits of visualizing stigma, thereby helping us to take measures for reducing stigma if an emerging infectious disease occurs in the future. Further, identifying the factors associated with stigma would contribute to our understanding of effective intervention methods such as information provision or counseling.

## Materials and methods

### Study design and participants

This was a cross-sectional study conducted as part of the Kanagawa Prospective “ME-BYO” Cohort Study (ME-BYO cohort) in Japan (26), which is one site of a collaborative genomic cohort study, namely the Japan Multi-Institutional Collaborative Cohort Study (J-MICC Study). Details of the J-MICC Study are described elsewhere (27). In short, the J-MICC Study is being conducted by 13 research groups in 12 prefectures in Japan using a standardized protocol. Apart from common standardized process, each research group is allowed to collect additional data for their own research purposes. At Kanagawa Cancer Center Research Institute (KCC), the baseline recruitment started in 2016 and the baseline survey is still ongoing in 2022. The participants of the ME-BYO cohort were people aged 20–85, and living or working in Kanagawa Prefecture, Japan.

The data were obtained from participants recruited from December 2020 to March 2021, from two sites: the Driver's License Examination Center of Kanagawa Prefecture in Yokohama city and a manufacturing company located in Hiratsuka city, Kanagawa, Japan. Passers-by near the Driver's License Examination Center of Kanagawa Prefecture were asked for voluntary cooperation after providing their informed consent. Registered residents from the Kanagawa prefecture appear at the Center regardless of their residential area in Kanagawa; therefore, the participants were diverse and representative from the whole prefecture to a certain degree. At the second site, employees were sent an invitation to participate in the study along with a request for informed consent. Recruitment was performed in combination with research to clarify the subclinical infection rate in the general population. Thus, persons without a history of COVID-19 were eligible. The history of infection was confirmed by self-report based on whether the participants had ever tested positive by polymerase chain reaction or antigen test for SARS-CoV-2. The timeline of the research is illustrated in Supplementary Figure 1.

A total of 1,573 participants in the ME-BYO cohort were recruited during the above period. Participants were instructed to respond to two questionnaires: (1) a baseline questionnaire for the genomic cohort study, and (2) a questionnaire to clarify the subclinical infection rate in the general population (additional baseline questionnaire); completion of these two questionnaires was mandatory for participation in the study. Furthermore, we also requested that participants fill out an optional web-based questionnaire on stigma related to COVID-19. Age, sex, socioeconomic status (income, education, and job rank), QOL, and psychological distress were obtained from the baseline questionnaire and used to assess the association with the COVID-19 stigma.

### Measurements

We measured stigma related to COVID-19 based on the Japanese version of the Cancer Stigma Scale (J-CASS) (28), which is a translated version of the original CASS consisting of 25 items (22). J-CASS was provided by researchers at the Center for Cancer Control and Information Services, National Cancer Center, Japan. The participants of the J-CASS study were selected from the general population with an age range of 20–69 years who could read Japanese (28). The scale comprises 25 items on a 6-point Likert scale (1: Strongly disagree to 6: Strongly agree) along with “not sure,” and the score is calculated by averaging the scores obtained. Respondents who answered “not sure” for more than 20% of the total answers (~30% of respondents) were excluded from the analysis (28). We adapted the CASS according to our hypothesis that we can measure stigma related to COVID-19 by replacing “cancer” with “COVID-19” in the CASS, based on previous research indicating that the underlying concept of stigma scales are common. However, the four items considered cancer-specific and unsuitable for evaluating COVID-19-related stigma simply by replacing the disease name were revised, as shown in Table 1. Furthermore, we added one question to reflect the wellknown phenomenon of intrafamily infection (Table 1). The final scale consisted of 26 items evaluated on a 6-point Likert scale (1 = strongly disagree to 6 = strongly agree), measuring stigma related to COVID-19 in six factors as it is in the CASS: means of the applicable items for Awkwardness, Severity, Avoidance, Policy Opposition, Personal Responsibility, and Financial Discrimination were calculated. We defined each factor as follows: Avoidance is an attitude of avoiding infected persons; Personal Responsibility refers to believing that the infected persons themselves are responsible for their infection; Severity refers to believing that a person cannot return to normal once infected; Policy Opposition is the expectation of more public funding investment for the patient's care; Awkwardness refers to feeling uncomfortable being with a person who had been infected and; Financial Discrimination refers to accepting putting a financial burden on infected people.

**Table 1:** List of items that were corrected, other than by replacing the disease name.

Items*	**Items in the cancer stigma scale**	**Items in our study**
**Avoidance**		
Item number 19 appendix	–	If a close friend or family had a COVID-19, I would try to avoid them (even if healed).
**Policy opposition**		
Item number 22	More government funding should be spent on the care and treatment of those with cancer.	More government funding should be spent on prevention measures against COVID-19.
Item number 23	We have a responsibility to provide the best possible care for people with cancer.	We have a responsibility to follow measures for the prevention of COVID-19.
**Financial discrimination**		
Item number 20	It is acceptable for banks to refuse to make loans to people with cancer.	It is acceptable to exclude people who had COVID-19 from financial support by the government.
Item number 24	Banks should be allowed to refuse mortgage applications for cancer-related reasons.	It is acceptable to exclude stores or facilities that caused COVID-19 from financial support by the government.

^*^Item numbers correspond to the item number in the validation paper of J-CASS (22).

J-CASS, Japanese version of the cancer stigma scale.

Information related to the attitude of the participants toward COVID-19 was obtained from the additional baseline questionnaire (e.g., Was someone close to you [family, colleague, classmate] infected with COVID-19? Do you think people who had COVID-19 lack morals?).

QOL was evaluated by EQ-5D-5L (EuroQoL 5- Dimension 5-Level) score (29, 30). EQ-5D-5L is a tool to assess health-related QOL in five dimensions (mobility, self-care, usual activities, pain/discomfort, and anxiety/depression), with five levels (no, slight, moderate, severe, and extreme problems). The score ranges from 0 to 1, where 1 indicates full health. Psychological distress was evaluated by the 6-item Kessler Psychological Distress Scale (K6) score, a robust non-specific psychological distress measurement tool (31, 32). K6 score is calculated from 6 items using a 5-Likert scale, with a total score ranging from 0 to 24; a higher score indicates more severe distress. We used a Japanese version of the scale (29, 31) translated and validated from the original scale developed in English (30, 32).

Age was categorized into five categories in the analysis (20–39, 40–49, 50–59, 60–69, and 70 years or older), annual household income was categorized into two groups (≤6, >6 million yen/year [~45 thousand US dollar]), and individual income was categorized into two groups (≤3, >3 million yen/year [~25 thousand US dollar]).

### Statistical analysis

All statistical analyses were performed with R (version 4.1.0; R Core Team, Vienna, Austria) (33). The reliability and validity of the COVID-19 stigma were checked in accordance with COnsensus-based Standards for the selection of health Measurement INstruments reporting guideline (34). We conducted a confirmatory factor analysis (CFA) using the cfa function in the R lavaan (version 0.6–9) package using a robust maximum likelihood model with oblique rotation (Promax), as was done in previous studies (22, 28, 35), assuming that stigma related to COVID-19 would have the same structure as the CASS and J-CASS. We included the correlation of the residual errors between items 5 and 8, 10 and 14, 13 and 16, 14 and 15, and 19 and 19a, as these questions had similar wording (refer to Table 2 for the item numbers). We could not include the correlation between items 10 and 11 because this would make the model impossible to identify. The model fit indices were calculated and evaluated with cut-off values to assess the goodness of fit as follows: Standardized Root Mean of the Residual (SRMR) < 0.08, Comparative fit index (CFI) > 0.95, Tucker–Lewis Index (TLI) > 0.95, and Root Mean Square Error of Approximation (RMSEA) < 0.06 (36).

**Table 2:** Explanatory factor analysis of COVID-19 stigma scale.

**Items**		Factor loadings*
**Avoidance**	
15	I would find it hard to talk to someone with COVID-19 (AW).	0.98
18	I would distance myself physically from someone with COVID-19.	0.97
19	If a colleague had COVID-19, I would try to avoid them (even if healed).	0.95
14	I would find it difficult being around someone with COVID-19 (AW).	0.93
19a	If a close friend or family had COVID-19, I would try to avoid them (even if healed).	0.85
16	I would feel irritated by someone with COVID-19.	0.81
12	I would try to avoid a person with COVID-19.	0.77
17	I would feel embarrassed discussing COVID-19 with someone who had it.	0.69
13	I would feel angered by someone with COVID-19.	0.66
20	It is acceptable to exclude people who had COVID-19 from financial support by the government. (FD)	0.44
**Personal responsibility**	
8	A person with COVID-19 is liable for their condition.	0.82
5	A person with COVID-19 is accountable for their condition.	0.65
9	If a person has COVID-19, it is probably their fault.	0.55
3	A person with COVID-19 is to blame for their condition.	0.49
**Severity**	
7	COVID-19 devastates the lives of those it touches.	0.93
4	Having COVID-19 usually ruins a person's career.	0.88
6	COVID-19 usually ruins close personal relationships.	0.69
1	Once you've had COVID-19, you can never be “normal” again.	0.65
2	Getting COVID-19 means having to mentally prepare oneself for death.	0.50
**Policy opposition**	
21	The needs of COVID-19 patients should be given top priority. (Reversed)	0.85
22	More government funding should be spent on the prevention measures against COVID-19. (Reversed)	0.74
23	We have a responsibility to follow the prevention measures for the prevention of COVID-19. (Reversed)	0.55
**Awkwardness**	
11	I would feel comfortable around someone with COVID-19 (Reversed)	0.89
10	I would feel at ease around someone with COVID-19 (Reversed)	0.86
Financial discrimination**	
24	It is acceptable to exclude stores or facilities that caused COVID-19 from financial support by the government.	-
25	It is acceptable for insurance companies to reconsider a policy if someone had COVID-19	-

(AW) items were included in the Awkwardness factor in the cancer stigma scale (CASS), while the (FD) item was included in financial discrimination in the CASS and Japanese version of the CASS.

^*^The highest factor loading for each item is shown in the relevant factor group. ^**^Financial discrimination factor was excluded from the analysis because all belonging items' factor loading was below 0.40.

Model fit was insufficient according to the results of the initial CFA (SRMR = 0.053, CFI = 0.897, TLI = 0.880, RMSEA = 0.083), thus, we performed exploratory maximum likelihood factor analysis using the fa function in the R psych (version 2.2.5) package to examine the structure of the scale, to check that the factors confirmed in the CASS are also appropriate for COVID-19 stigma (37). We checked the suitability of the data for structure detection in the factor analysis using the Kaiser-Meyer-Olkin (KMO) sampling adequacy measure and Bartlett's sphericity test using the KMO and cortest.bartlett function in the psych package, respectively (37). We excluded items with low factor loadings (< 0.4). The internal reliability of each factor was evaluated using Cronbach's alpha with a cut-off value of > 0.70, indicating satisfactory internal reliability (38). We could not assess test-retest reliability as the data were collected *via* the cross-sectional baseline survey of the cohort study. We then conducted CFA again according to the result of exploratory factor analysis.

In addition, the model was extensively analyzed by SEM using the sem function in the lavaan package, to further test our hypothesis that COVID-19 stigma affects QOL and K6 scores, assuming COVID-19 stigma as a latent variable consisted from the confirmed five factors also as latent variables (35): the factors identified by CFA were used as latent variables consisting of each item as an observed variable, and we assumed the latent variable of COVID-19 stigma using five factors as subscales. QOL and K6 were standardized by arcsine and square root transformation, respectively, using the bestNormalize function in the bestNormalize (version 1.8.2) package (39). The factors identified by factor analysis were used as latent variables consisting of each item as an observed variable, and we assumed the latent variable of COVID-19 stigma using them as subscales. Based on the modification indices, an additional correlation of the residual error between Avoidance and Awkwardness, and QOL and K6 was allowed.

The difference in COVID-19 stigma according to the subgroups of sex, age, socioeconomic status, and groups based on the questionnaire were compared for each factor. Scores of each factor were calculated as a mean of the items that belonged to each factor, and comparison was performed using a Kruskal-Wallis rank sum test, after checking normality using the Shapiro-Wilk test and graphically using the histogram and quintile-quintile plot. *Post-hoc* analysis was performed using the Bonferroni-corrected Dunn test if the *P*-value of the Kruskal-Wallis test was below 0.05 for the variables with more than three categories.

### Ethical approval

All research procedures were approved by the KCC ethics committee (28KEN-36, 2020EKI-79). Written informed consent was obtained from all participants for the ME-BYO cohort and the research to clarify subclinical infection rates in the general population, respectively.

### Results

Among the 1,573 participants, 257 (16.3%) answered the questionnaire on stigma related to COVID-19. There were no missing values in the questionnaire. Bar plots showing the proportion of answers to each item are shown in Supplementary Figure 2, and there were no items with extremely skewed responses. The mean age (standard deviation [SD]) was 54.5 (14.4), and 129 participants were male (50.2%). The mean age (SD) in males and females was 56.8 (15.4) and 52.2 (12.9), respectively. Twenty-three participants (11.2%) responded that someone close to them (family member, colleague, or classmate) had been infected with COVID-19.

The model fit indices obtained in the CFA to assess the structural validity were as follows: SRMR = 0.048, CFI = 0.963, TLI = 0.957, RMSEA = 0.053. All four indices met the criteria to assess the goodness of fit. The structure of the model was obtained from the results from the exploratory factor analyses, shown below.

**Figure 1:**
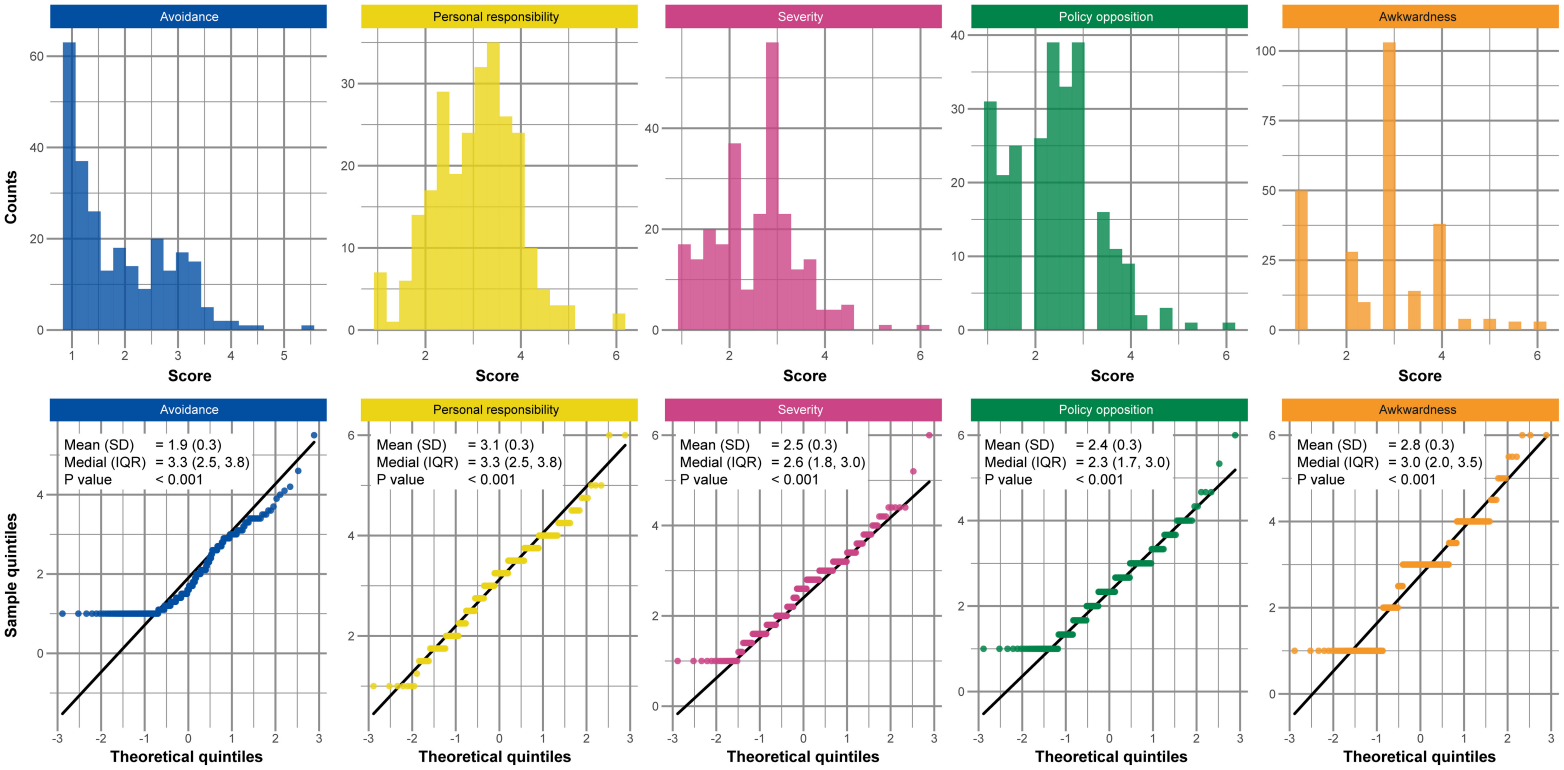
Histograms and QQ-plots of the scores for each factor of social stigma related to COVID-19. Definitions of each factor are as follows: avoidance is an attitude of avoiding the patient; personal responsibility is to anticipate that the infected persons themselves are responsible for their infection; severity is to anticipate that you could not return to normal again once infected; policy opposition is to expect more public funding investment for patients' care; awkwardness is an attitude of feeling uncomfortable being with a person who had the infection before. the *p*-values were calculated using the Shapiro-Wilk test. SD; standard deviation, IQR; inter-quartile range, QQ; quintile-quintile, COVID-19; coronavirus disease.

The results of the exploratory factor analysis are shown in Table 2 and Supplementary Table 1. The overall measure of sampling adequacy (KMO index) was 0.91, and the chi-square test statistic was 4,700.6 (*p*-value < 0.0001) in Bartlett's test of sphericity, indicating the suitability of the data. Factor loadings for two items in Financial Discrimination were below 0.4 (item numbers 24 and 25) and thus excluded from the analysis. As a result, stigma related to COVID-19 was evaluated with five factors, which explained 62.2% of the variance. Factor loading of each item is shown in Table 2. Three items belonged to a different factor in CASS; two items in Awkwardness (item numbers 14 and 15) and one item in Financial Discrimination (item number 20) in the CASS belonged to the Avoidance in COVID-19 stigma. Final scores for each factor and the result of the normality assessment are shown in Figure 1; all factors were non-normally distributed.

The correlation coefficient matrix of the five factors is shown in Table 3. The highest correlation between factors was observed for Avoidance and Awkwardness (*r* = 0.67). Cronbach's alpha for the total scale and each factor are also shown in Table 3, and all values met the criteria.

**Table 3:** Correlation coefficient matrix and internal consistency of each factor.

**Factors**	**F1**	**F2**	**F3**	**F4**	**F5**	**Total**
**Correlation**						
F1: Avoidance	1.00					
F2: Personal Responsibility	0.11	1.00				
F3: Severity	−0.51	−0.004	1.00			
F4: Policy opposition	0.52	0.05	−0.23	1.00		
F5: Awkwardness	0.67	−0.07	−0.34	0.50	1.00	
Internal consistency*	0.95 (0.94–0.96)	0.75 (0.70–0.80)	0.85 (0.82–0.88)	0.74 (0.69–0.80)	0.95 (0.94–0.96)	0.92 (0.91–0.94)

^*^Cronbach's alpha. The numbers in the parenthesis show the 95% confidence interval.

**Figure 2:**
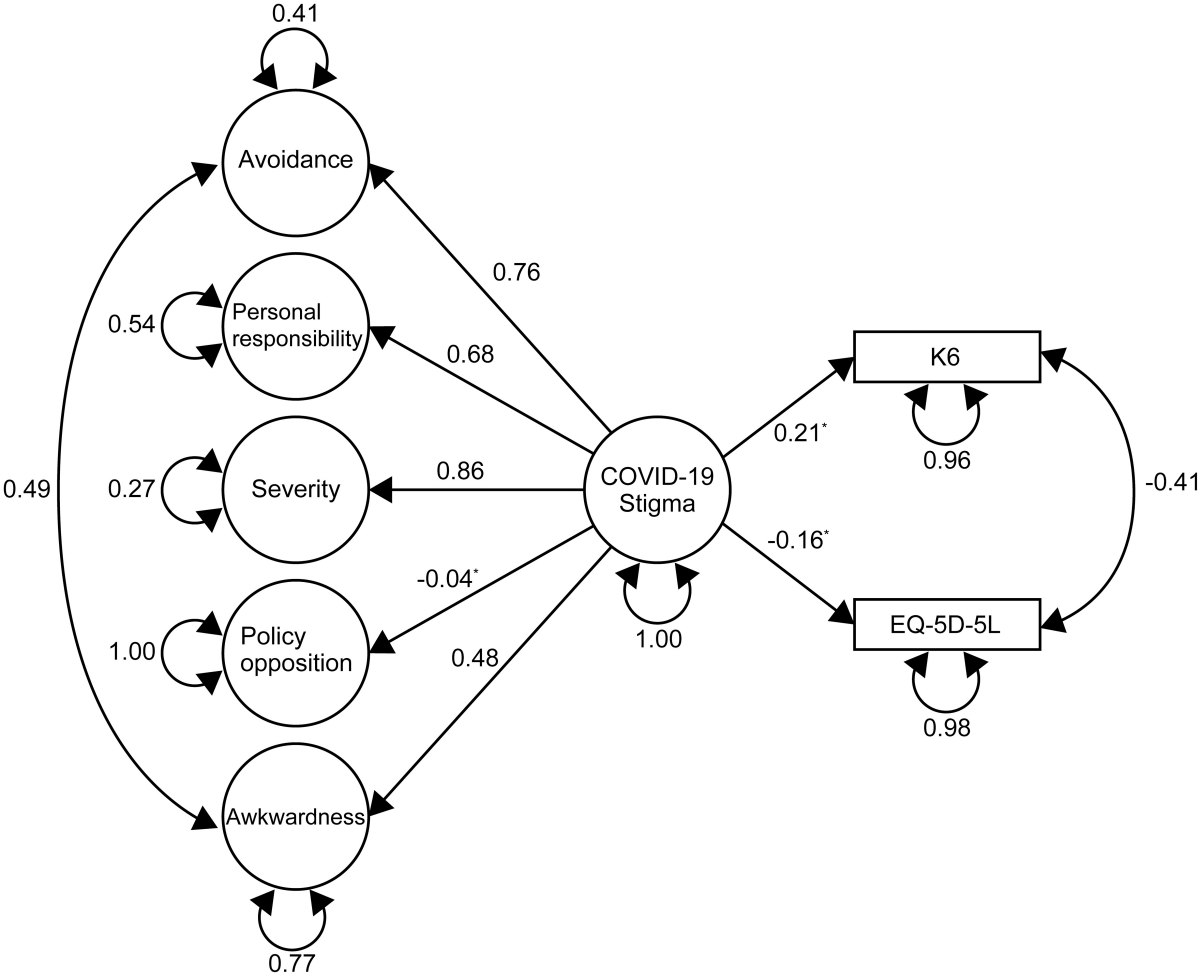
Path diagram and results of the structural equation modeling. Values next to each path indicate the standardized estimates. The double-headed curved arrows indicate the correlation of residual errors between the variables. The circular curved arrows represent the variance of error. Standardized Root Mean of the Residual = 0.055, Comparative fit index = 0.956, Tucker–Lewis Index = 0.951, Root Mean Square Error of Approximation= 0.053. *The *p*-values were < 0.0001 for all estimates except for Policy Opposition (*P* = 0.593), EQ-5D-5L (*P* = 0.023), and K6 (*P* = 0.002). COVID-19 stigma; social stigma related to the coronavirus disease, K6; 6-item Kessler Psychological Distress Scale, EQ-5D-5L; EuroQoL 5- Dimension 5-Level. COVID-19 stigma scale scores for each factor according to each characteristic subgroup. The values indicate the median (interquartile range). COVID-19; coronavirus disease 2019.

The result of the SEM is shown in Figure 2. Highest standardized coefficient for the COVID-19 stigma (latent variable) was Severity (beta = 0.86). Regression coefficients of K6 and EQ-5D-5L on COVID-19 stigma were 0.21 (95% CI 0.074–0.342) and −0.159 (95% CI −0.295–−0.022). Other details of the results are shown in Supplementary Table 2. Model fit indices for the SEM were as follows; SRMR = 0.055, CFI = 0.956, TLI = 0.951, RMSEA = 0.053.

Table 4 shows the results of the Kruskal-Wallis test. In Policy Opposition and Awkwardness, *p*-value for the mean difference in age was below the cut-off of 0.05. Avoidance in people aged 70 years or older also seemed to be high, although *p*-value was above the cut-off. *Post-hoc* analysis indicated that scores for Policy Opposition in people aged 20–39 years old were higher from those of 60–69 years old (*p*-value = 0.037), and score for Awkwardness in people aged ≥ 70 years were higher from those of 20–39 years old (*p*-value = 0.026), 40–49 years old (*p*-value = 0.005), and 50–59 years old (*p*-value = 0.035). The median score (inter-quartile range) for Severity in males was 2.40 (1.60, 3.00), and 2.80 (2.00, 3.20) in females (*p*-value = 0.006). COVID-19 stigma score was higher for those who felt anxiety regarding the transmission or spread of COVID-19 and those who did not, especially for Avoidance and Severity (*p*-value < 0.001). The score for Awkwardness was higher in people who answered that they do not understand risky behaviors that are likely to lead to the transmission or spread of COVID-19 (*p*-value = 0.028).

**Table 4:** COVID-19 stigma scale scores for each factor according to each characteristic subgroup.

	***n***	**Avoidance**	**Personal responsibility**	**Severity**	**Policy opposition**	**Awkwardness**
**Sex**						
Male	129	1.50 (1.00, 2.60)	3.25 (2.50, 3.50)	2.40 (1.60, 3.00)	2.33 (1.67, 3.00)	3.00 (2.00, 3.00)
Female	128	1.65 (1.10, 2.70)	3.25 (2.75, 3.75)	2.80 (2.00, 3.20)	2.33 (1.67, 3.00)	3.00 (2.00, 3.50)
*p*-value		0.465	0.086	0.006*	0.846	0.408
**Age group (years)**						
20–39	43	1.80 (1.15, 2.65)	3.50 (2.62, 3.75)	2.80 (2.00, 3.00)	2.67 (2.33, 3.17)	3.00 (2.00, 3.00)
40–49	48	1.40 (1.00, 2.22)	3.12 (2.50, 3.75)	2.80 (1.60, 3.20)	2.33 (1.67, 3.00)	3.00 (1.00, 3.00)
50–59	76	1.50 (1.00, 2.70)	3.25 (2.75, 3.75)	2.70 (1.80, 3.05)	2.33 (1.67, 3.00)	3.00 (2.00, 3.50)
60–69	51	1.50 (1.15, 2.10)	3.00 (2.50, 3.50)	2.40 (2.00, 3.00)	2.33 (1.50, 2.83)	3.00 (2.00, 3.00)
70–85	39	2.10 (1.35, 3.00)	3.25 (2.38, 3.75)	2.60 (1.70, 3.20)	2.33 (1.50, 3.00)	3.00 (3.00, 4.00)
*p*-value		0.064	0.282	0.974	0.041*	0.007*
**Annual household income**						
≤ 6 million yen	135	1.50 (1.10, 2.30)	3.00 (2.50, 3.50)	2.60 (1.80, 3.00)	2.33 (1.67, 3.00)	3.00 (2.50, 3.00)
> 6 million yen	109	1.60 (1.00, 2.70)	3.25 (2.25, 3.75)	2.60 (1.80, 3.20)	2.33 (1.67, 3.00)	3.00 (1.00, 3.50)
*p*-value		0.750	0.573	0.584	0.470	0.098
**Individual income**						
≤ 3 million yen	131	1.50 (1.10, 2.60)	3.25 (2.50, 3.75)	2.80 (2.00, 3.00)	2.33 (1.67, 3.00)	3.00 (2.00, 3.00)
> 3 million yen	121	1.70 (1.10, 2.70)	3.25 (2.50, 3.75)	2.40 (1.60, 3.20)	2.33 (1.67, 3.00)	3.00 (2.00, 3.50)
*p*-value		0.718	0.894	0.110	0.395	0.779
**Education**						
High school graduate or earlier	50	1.40 (1.10, 2.10)	3.25 (2.56, 3.50)	2.50 (1.80, 3.00)	2.33 (1.67, 3.00)	3.00 (2.62, 3.38)
Junior college/technical school graduate	72	2.00 (1.10, 2.75)	3.25 (2.75, 3.81)	2.80 (2.00, 3.20)	2.33 (1.67, 3.00)	3.00 (2.00, 3.50)
University/graduate school graduate	134	1.70 (1.00, 2.68)	3.25 (2.50, 3.75)	2.60 (1.85, 3.20)	2.33 (1.67, 3.00)	3.00 (2.00, 3.38)
*p*-value		0.199	0.240	0.375	0.859	0.467
**Job rank**						
Manager	22	1.20 (1.00, 1.65)	2.75 (2.25, 3.50)	1.80 (1.20, 2.60)	2.33 (1.33, 3.50)	2.25 (1.00, 3.00)
Permanent employee	82	1.70 (1.02, 2.70)	3.25 (2.50, 3.75)	2.60 (1.80, 3.20)	2.33 (1.75, 3.00)	3.00 (2.00, 3.00)
Public officers	15	1.50 (1.20, 2.05)	3.50 (3.00, 3.75)	2.40 (1.90, 2.90)	2.33 (1.83, 3.00)	3.00 (1.50, 3.00)
Contractor/temporary	20	1.90 (1.28, 3.40)	3.12 (2.25, 4.00)	2.80 (1.95, 3.20)	2.50 (1.33, 3.00)	3.25 (2.75, 4.00)
Part-time	41	1.60 (1.20, 2.30)	3.25 (2.75, 3.75)	2.80 (2.00, 3.00)	2.33 (1.33, 3.00)	3.00 (2.00, 3.50)
Homemaker	24	2.05 (1.00, 2.82)	3.25 (2.75, 3.75)	2.70 (2.20, 3.05)	2.33 (1.92, 2.75)	3.00 (1.75, 3.00)
Retired	18	1.75 (1.05, 2.90)	2.88 (1.69, 3.50)	2.60 (1.70, 3.20)	2.17 (1.08, 3.00)	3.00 (2.62, 4.00)
Students	4	1.95 (1.25, 2.65)	3.50 (3.06, 3.50)	2.80 (2.40, 3.15)	3.00 (2.83, 3.25)	3.00 (2.75, 3.12)
*p*-value		0.513	0.442	0.136	0.626	0.056
**COVID-19 in someone close (family, colleague, schoolmate)**						
No	234	1.70 (1.10, 2.70)	3.25 (2.50, 3.75)	2.60 (1.80, 3.00)	2.33 (1.67, 3.00)	3.00 (2.00, 3.50)
Yes	23	1.40 (1.05, 2.05)	3.25 (2.12, 3.62)	2.60 (1.80, 3.20)	2.67 (2.33, 3.00)	3.00 (2.00, 3.00)
*p*-value		0.287	0.610	0.834	0.049*	0.288
**People who had COVID-19 lack morals**						
No	214	1.60 (1.00, 2.60)	3.25 (2.31, 3.50)	2.60 (1.80, 3.00)	2.33 (1.67, 3.00)	3.00 (2.00, 3.00)
Yes	43	1.70 (1.20, 3.00)	3.50 (2.88, 4.00)	2.80 (1.90, 3.20)	2.33 (1.67, 3.00)	3.00 (2.00, 4.00)
*p*-value		0.106	0.007*	0.433	0.993	0.155
**People who had COVID-19 lack common sense**						
No	231	1.60 (1.10, 2.70)	3.25 (2.50, 3.75)	2.60 (1.80, 3.10)	2.33 (1.67, 3.00)	3.00 (2.00, 3.25)
Yes	26	1.55 (1.12, 2.58)	3.50 (3.06, 4.00)	2.60 (1.80, 3.00)	2.00 (1.67, 2.67)	3.00 (2.62, 3.50)
*p*-value		0.945	0.034*	0.714	0.184	0.366
**Do you understand risky behaviors that are likely to lead to the transmission or spread of COVID-19?**						
No	41	1.60 (1.10, 2.70)	3.25 (2.50, 4.00)	2.80 (1.80, 3.00)	2.33 (1.67, 3.00)	3.00 (2.50, 4.00)
Yes	216	1.60 (1.00, 2.62)	3.25 (2.50, 3.75)	2.60 (1.80, 3.20)	2.33 (1.67, 3.00)	3.00 (2.00, 3.00)
*p*-value		0.586	0.206	0.682	0.902	0.028*
**Are you taking action to prevent the transmission and spread of COVID-19 every day?**						
No	23	1.80 (1.00, 2.55)	3.50 (2.50, 3.75)	2.60 (2.20, 2.80)	2.33 (1.83, 3.17)	3.00 (1.50, 3.00)
Yes	234	1.60 (1.10, 2.68)	3.25 (2.50, 3.75)	2.60 (1.80, 3.20)	2.33 (1.67, 3.00)	3.00 (2.00, 3.50)
*p*-value		0.742	0.256	0.644	0.392	0.302
**Do you feel anxiety associated with the transmission or spread of COVID-19?**						
No	37	1.10 (1.00, 1.50)	2.50 (1.75, 3.50)	1.80 (1.40, 2.20)	3.00 (2.00, 4.00)	2.50 (1.00, 3.00)
Yes	220	1.80 (1.10, 2.70)	3.25 (2.75, 3.75)	2.80 (2.00, 3.20)	2.33 (1.67, 3.00)	3.00 (2.00, 3.50)
*p*-value		<0.001*	0.005*	<0.001*	0.001*	0.030*

The values indicate the median (interquartile range). COVID-19; coronavirus disease.

^*^These p-values were below the cut-off of 0.05.

## Discussion

This is the first study to elucidate the characteristics of population groups prone to stigma and factors associated with the stigma, using data collected during the COVID-19 pandemic. The results suggest that individuals aged ≥70 years more likely to exhibit COVID-19 stigma. In addition, COVID-19 stigma was shown to be associated with QOL and psychological distress, even in uninfected individuals.

Factor analysis indicated that COVID-19 stigma consists of five factors: Avoidance, Personal Responsibility, Severity, Policy Opposition, and Awkwardness. These results highlighted the internal consistency and structural validity of the scale; however, we could not assess the reliability and measurement error. These results are consistent with the CASS, except for Financial Discrimination which was not evident for COVID-19 stigma. As restrictions due to the COVID-19 and the economic burden caused were practically equivalent among populations, many people might have perceived that financial support is decisive, reducing the factor loading for Financial Discrimination in the factor analysis (Table 2).

COVID-19 stigma score for Avoidance and Awkwardness was higher for individuals aged ≥ 70 years. COVID-19 patients older than 70 years old are at risk for severe illness (1, 3, 40). Age and other risk factors such as underlying medical conditions are known to be associated with severe outcomes or death (40, 41). In addition, concerns about unrecognized transmission from the pre- or pauci-symptomatic patients were especially strong among higher risk people, due to difficulty in preventing such infections (17). Higher scores in Avoidance and Awkwardness in individuals over 70 could be a result reflective of the above aspects. In addition, association between age and stigma related to other diseases, such as HIV/AIDS, and age itself, cause prejudice known as ageism (42). However, we were unable to distinguish the association between age and other risk factors of the disease that might correlate with age, as age was always an alternate endpoint. Disease risk of COVID-19 was increased for higher ages. Nevertheless, in future emerging infectious diseases where younger age is associated with higher risk, stigma score might not be associated with age, but instead with other risk factors associated with the disease.

Among the five factors of COVID-19 stigma, the distribution of the score was different in Policy Opposition which consisted of items related to public funding (Table 4). The score for Policy Opposition was higher in younger individuals, who might be hesitant to put public funding, such as loan system, support funding, or financial aid, into the economic and social consequences caused by COVID-19 (43). In Japan, social security expenses continue to increase due to the declining birthrate and aging population, resulting in an imbalance in benefits and burden between generations (44, 45). The heaviest burden is placed on citizens who recently joined the workforce and hence begun paying taxes and those struggling to make ends meet due to childbirth and childcare (44, 45). This may explain why the score for Policy Opposition was higher in younger people likely to be uncompromising about the usage of public funds consisting of taxes.

Severity had the biggest effect on COVID-19 stigma (Figure 2), suggesting that anxiety and fear surrounding the consequences caused by getting infected is a crucial component of COVID-19 stigma. Severity is based on a dreadful image of the disease and society's attitudes, along with the assumption that life will be disrupted by COVID-19 (Table 2). During the pandemic, there has been an abundance of information regarding clusters, the prognosis of critically ill patients, individuals suffering from the aftereffects and economically, and fake news that cause insecurity, all of which have exacerbated Severity (17, 46). Thus, to reduce Severity, it is crucial to assure and show that one can return to social life once recovered. To achieve this, we propose providing opportunities, especially to populations exhibiting more bias such as individuals aged ≥70 years, to promote active communication with a person who experienced COVID-19 and returned to their normal life. Being in contact with a person who had been infected is more effective than just an educational intervention (19, 47). However, indicating an optimal educational intervention was difficult within this study, and therefore further research is required.

Differences in COVID-19 stigma were observed between participants who felt anxiety associated with infection and spread of COVID-19 and who did not (Table 4). Associated *p*-values were comparatively higher for Awkwardness, while the Awkwardness score was higher for participants who answered that they did not understand the risky behaviors that are likely to lead to the transmission or spread of COVID-19. Thus, the COVID-19 stigma scored in this study did not just reflect the disinterest of participants but was associated with anxiety and lack of knowledge. Taken together, individuals whose Awkwardness score is high would be candidates for an intervention aiming to expand their knowledge, and as noted above, people with a high Severity score could be candidates for an intervention aiming to taper their anxiety. Furthermore, COVID-19 stigma was shown to have a negative effect on QOL and psychological distress (Table 4; Figure 2). The interventions aiming to reduce Awkwardness and Severity could also contribute to improvements in QOL and psychological distress. There have been an increasing number of suicides associated with the COVID-19 pandemic (43, 48). Intervention aiming to reduce the COVID-19 stigma may also contribute to suicide prevention by the ripple effect on QOL and mental status.

The current approach to reduce COVID-19 stigma was undertaken by disseminating a message asking for an end to discrimination and prejudice against people infected with COVID-19 without targeting a specific population (10). We assume that the high-risk approach, rather than the population approach taken currently, would have merit on the strategy to reduce the social stigma including the COVID-19 stigma as well as lifestyle related diseases (9, 49). According to the results of this study, priority targets for an efficient intervention would be individuals older than 70 years, who have a higher chance of exhibiting more bias toward people infected by COVID-19. Also, the Severity score was slightly higher in females than males; the difference in median was 0.40 points (*p*-value = 0.006). Thus, females may be more apprehensive about getting infected, although the absolute difference and the strength of evidence were small. However, the incidence of depression in females is twice as high as in males due to stress caused by life events, partly due to biological differences between sexes (50). Higher scores for Severity in females may result from females being more apprehensive about the diverse disruption caused by the COVID-19 pandemic.

### Limitations

This study has some limitations. First, the scale used to measure the COVID-19 stigma was not validated in advance. Developing and validating a new scale for the COVID-19 stigma takes time, and thus we attempted to measure COVID-19 stigma using a validated stigma scale in Japanese for cancer (J-CASS) by changing the disease name (22, 28). Nevertheless, our findings demonstrated that the validity measures of the score were satisfactory, suggesting that the scale could be perceived as indicating COVID-19 stigma. Meanwhile, as the study was undertaken in conjunction with a baseline survey of a cohort study, the analysis was cross-sectional which also limited us to assess the reliability of the stigma scale. Moreover, as responding to the survey regarding COVID-19 stigma was optional, only 16.3% of the target population responded and the results are biased by selection. In addition, because our study participants were limited to people aged 20–85, our study population was ~7 years higher in age compared to the general population, which indicates the existence of a selection bias. Therefore, the results cannot be generalized to people outside this range.

### Suggestions for future studies

We intend to further assess the relationship between COVID-19 stigma and mental wellbeing, particularly the opposite relationship and degree of impact on QOL and psychological stress from COVID-19 stigma. With regards to measurements, since COVID-19 is a wellknown infectious disease which triggered a global pandemic, our approach for measuring disease-related stigma needs to be validated in other diseases, such as rare diseases or infectious diseases with lower infectious capability for generalizability. Lastly, future studies to elucidate an optimal intervention aiming to ameliorate stigma are required. For example, we could conduct a study to evaluate the effects of an intervention such as those which provide an opportunity to communicate with a person who has experienced COVID-19 in the population exhibiting more stigma.

## Conclusion

Older individuals, who exhibit a higher risk of getting infected with COVID-19, are likely to exhibit greater prejudice against COVID-19. Furthermore, COVID-19 stigma was shown to have a negative effect on QOL and psychological distress even for uninfected populations.

## Data availability statement

Data cannot be shared publicly due to ethical restrictions. Data described in the manuscript will be made available upon application and approval from the ME-BYO cohort office (contact via the corresponding author) for researchers who meet the criteria for data sharing.

## Ethics statement

The studies involving human participants were reviewed and approved by Kanagawa Cancer Center. The patients/participants provided their written informed consent to participate in this study.

## Author contributions

ES and SN conceptualized and designed the research and wrote the first draft of the manuscript.. SN and HN designed the ME-BYO cohort. SN, KW, HI, NS, YS, and HN conducted the ME-BYO cohort and provided data. SN was responsible for data curation and data analysis. KW, KT, and HN were involved in interpretation of results. All authors read and approved the final manuscript.

## Funding

This study was supported by the Japan Society for the Promotion of Science (JSPS) KAKENHI Grant [No. 16H06277 (CoBiA)] from the Japanese Ministry of Education, Culture, Sports, Science and Technology. This work was also supported in part by a grant from the Kanagawa Prefectural Government of Japan.

## Conflict of interest

Authors HI and NS are members of Hygeia Communication General Incorporated Association. The remaining authors declare that the research was conducted in the absence of any commercial or financial relationships that could be construed as a potential conflict of interest.

## Publisher's note

All claims expressed in this article are solely those of the authors and do not necessarily represent those of their affiliated organizations, or those of the publisher, the editors and the reviewers. Any product that may be evaluated in this article, or claim that may be made by its manufacturer, is not guaranteed or endorsed by the publisher.
