# Supplementary material for: COVID-19-related stigma and its relationship with mental wellbeing: A cross-sectional analysis of a cohort study in Japan
Source: Front Public Health. 2022 Sep 29;10:1010720. doi: 10.3389/fpubh.2022.1010720 (PMC9558281; doi:10.3389/fpubh.2022.1010720)
Supplement: Supplementary file 1 [file Data_Sheet_1.PDF]

Supplementary Material

1 Supplementary Figures and Tables

1.1 Supplementary Figures

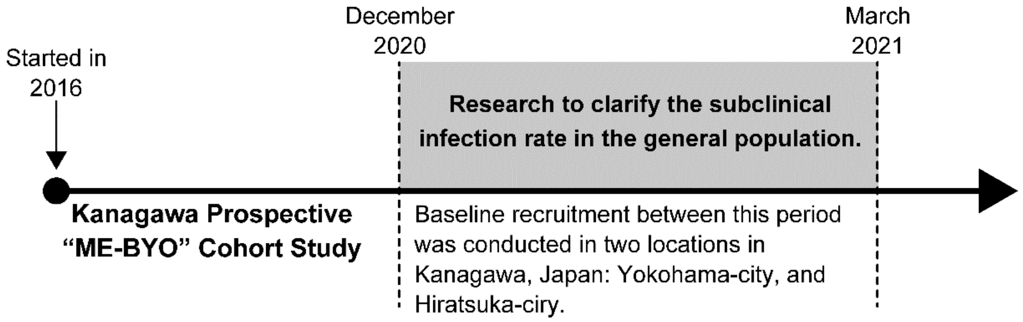

Supplementary Figure 1. Research timeline

The location at Yokohama city was adjacent to the Kanagawa Cancer Center Research Institute. Passers-by near the Driver's License Examination Center of Kanagawa Prefecture were requested for voluntary cooperation after providing informed consent. The location at Hiratsuka city was at a manufacturing company where workers were sent an invitation to participate, accompanied by a request for informed consent.

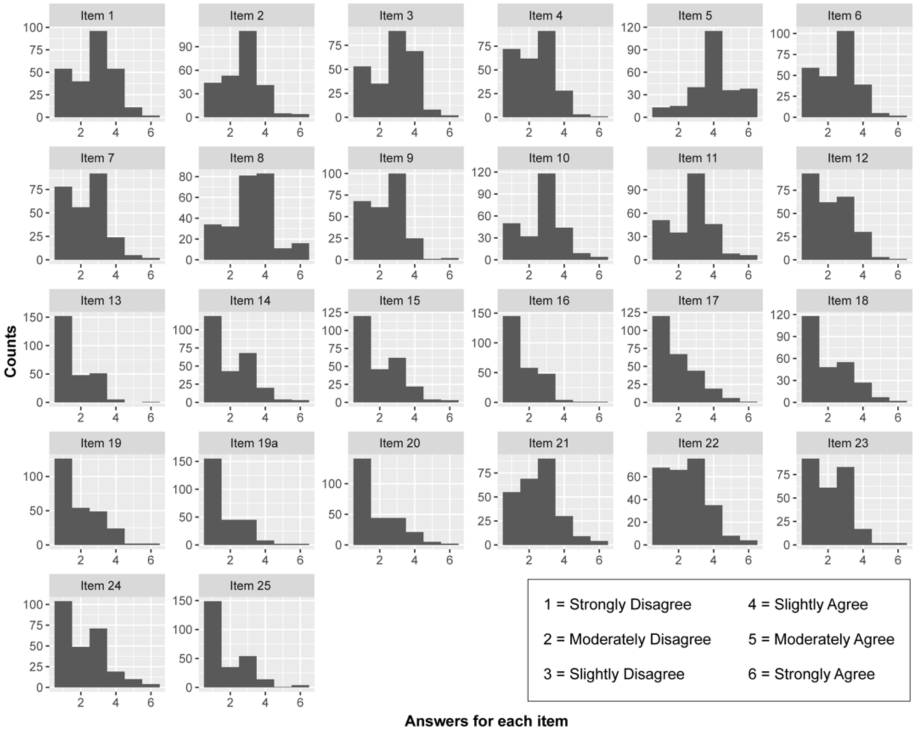

Figure S2. Barplots of the answers for each item

1.2 Supplementary Table

Table S1. Full result of the explanatory factor analysis of COVID-19 stigma scale

Items		Factor Loadings				
		Avoidance	Personal Responsibility	Severity	Policy Opposition	Awkwardness
**Avoidance**						
15	I would find it hard to talk to someone with COVID-19 (AW).	0.98	-0.19	0.05	-0.06	-0.02
18	I would distance myself physically from someone with COVID-19.	0.97	-0.05	-0.08	-0.01	0.09
19	If a colleague had COVID-19, I would try to avoid them (even if healed).	0.95	-0.03	0.00	-0.01	0.00
14	I would find it difficult being around someone with COVID-19 (AW).	0.93	-0.15	0.01	-0.10	0.05
19a	If a close friend or family had COVID-19, I would try to avoid them (even if healed).	0.85	0.09	-0.08	0.00	0.00
16	I would feel irritated by someone with COVID-19.	0.81	0.07	0.04	0.02	-0.11
12	I would try to avoid a person with COVID-19.	0.77	-0.07	0.03	-0.03	0.20
17	I would feel embarrassed discussing COVID-19 with someone who had it.	0.69	0.04	0.03	-0.01	0.02
13	I would feel angered by someone with COVID-19.	0.66	0.14	0.11	0.01	-0.11
20	It is acceptable to exclude people who had COVID-19 from financial support by the government. (FD)	0.44	0.11	-0.03	0.15	0.01
**Personal Responsibility**						
8	A person with COVID-19 is liable for their condition.	-0.07	0.82	-0.11	0.03	0.11
5	A person with COVID-19 is accountable for their condition.	-0.04	0.65	-0.14	-0.09	0.03
9	If a person has COVID-19, it is probably their fault.	0.09	0.55	0.23	0.10	-0.07
3	A person with COVID-19 is to blame for their condition.	0.05	0.49	0.20	0.01	-0.06
**Severity**						
7	COVID-19 devastates the lives of those it touches.	-0.01	-0.13	0.93	0.06	-0.04
4	Having COVID-19 usually ruins a person’s career.	0.07	-0.08	0.88	-0.01	-0.11
6	COVID-19 usually ruins close personal relationships.	0.00	0.02	0.69	0.08	0.13
1	Once you’ve had COVID-19, you can never be “normal” again.	0.00	-0.06	0.65	-0.02	0.13
2	Getting COVID-19 means having to mentally prepare oneself for death.	0.00	0.16	0.50	-0.16	0.01
**Policy Opposition**						
21	The needs of COVID-19 patients should be given top priority. (Reversed)	-0.04	0.02	-0.09	0.85	0.11
22	More government funding should be spent on the prevention measures against COVID-19. (Reversed)	-0.02	0.04	-0.04	0.74	-0.06

23	We have a responsibility to follow the prevention measures for the prevention of COVID-19. (Reversed)	0.01	-0.09	0.08	0.55	-0.02
**Awkwardness**						
11	I would feel comfortable around someone with COVID-19 (Reversed)	0.05	0.07	0.05	0.04	0.86
10	I would feel at ease around someone with COVID-19 (Reversed)	0.11	0.04	0.03	-0.03	0.89

(AW) items were included in the Awkwardness factor in the cancer stigma scale (CASS), while the (FD) item was included in financial discrimination in the CASS and Japanese version of the CASS.

Table S2. Details of the results from structural equation modeling

Left-hand side variable	Operator*	Right-hand side variable	Estimate	SE	p-value	95% confidence interval
Avoidance	=~	Item number 12	0.881	0.016	< 0.0001	(0.851–0.912)
Avoidance	=~	Item number 13	0.700	0.033	< 0.0001	(0.634–0.765)
Avoidance	=~	Item number 14	0.866	0.017	< 0.0001	(0.833–0.9)
Avoidance	=~	Item number 15	0.883	0.015	< 0.0001	(0.853–0.913)
Avoidance	=~	Item number 16	0.785	0.026	< 0.0001	(0.735–0.835)
Avoidance	=~	Item number 17	0.737	0.030	< 0.0001	(0.678–0.796)
Avoidance	=~	Item number 18	0.944	0.009	< 0.0001	(0.927–0.961)
Avoidance	=~	Item number 19	0.934	0.010	< 0.0001	(0.914–0.953)
Avoidance	=~	Item number 19a	0.814	0.023	< 0.0001	(0.77–0.859)
Avoidance	=~	Item number 20	0.489	0.049	< 0.0001	(0.393–0.586)
Personal_responsibility	=~	Item number 3	0.702	0.043	< 0.0001	(0.618–0.786)
Personal_responsibility	=~	Item number 5	0.333	0.063	< 0.0001	(0.21–0.457)
Personal_responsibility	=~	Item number 8	0.562	0.051	< 0.0001	(0.462–0.662)
Personal_responsibility	=~	Item number 9	0.870	0.037	< 0.0001	(0.797–0.942)
Severity	=~	Item number 1	0.688	0.038	< 0.0001	(0.614–0.762)
Severity	=~	Item number 2	0.587	0.046	< 0.0001	(0.497–0.676)
Severity	=~	Item number 4	0.838	0.025	< 0.0001	(0.789–0.886)
Severity	=~	Item number 6	0.737	0.034	< 0.0001	(0.672–0.803)
Severity	=~	Item number 7	0.832	0.025	< 0.0001	(0.782–0.881)
Policy_opposition	=~	Item number 21	0.874	0.053	< 0.0001	(0.77–0.977)
Policy_opposition	=~	Item number 22	0.727	0.052	< 0.0001	(0.626–0.829)
Policy_opposition	=~	Item number 23	0.528	0.055	< 0.0001	(0.42–0.636)
Awkwardness	=~	Item number 10	0.910	0.020	< 0.0001	(0.871–0.948)
Awkwardness	=~	Item number 11	0.998	0.018	< 0.0001	(0.963–1.033)
Item number 5	~~	Item number 8	0.471	0.052	< 0.0001	(0.37–0.573)
Item number 13	~~	Item number 16	0.679	0.035	< 0.0001	(0.61–0.748)
Item number 14	~~	Item number 15	0.584	0.045	< 0.0001	(0.496–0.673)
Item number 14	~~	Item number 10	-0.151	0.053	0.005	(-0.255–-0.046)
Item number 19	~~	Item number 19a	0.311	0.067	< 0.0001	(0.179–0.443)
Avoidance	~~	Awkwardness	0.492	0.065	< 0.0001	(0.366–0.619)
COVID-19 stigma	=~	Avoidance	0.760	0.047	< 0.0001	(0.668–0.851)
COVID-19 stigma	=~	Personal_responsibility	0.676	0.055	< 0.0001	(0.568–0.784)
COVID-19 stigma	=~	Severity	0.864	0.048	< 0.0001	(0.771–0.957)
COVID-19 stigma	=~	Policy_opposition	-0.042	0.078	0.593	(-0.195–0.111)
COVID-19 stigma	=~	Awkwardness	0.473	0.063	< 0.0001	(0.35–0.597)
EQ-5D-5L	~	COVID-19 stigma	-0.159	0.070	0.023	(-0.295–-0.022)
K6	~	COVID-19 stigma	0.208	0.069	0.002	(0.074–0.342)
Q101_stg	~~	Q101_stg	0.527	0.052	< 0.0001	(0.425–0.629)
Q102_stg	~~	Q102_stg	0.656	0.054	< 0.0001	(0.551–0.761)
Q103_stg	~~	Q103_stg	0.507	0.060	< 0.0001	(0.389–0.626)
Q104_stg	~~	Q104_stg	0.298	0.042	< 0.0001	(0.217–0.38)
Q105_stg	~~	Q105_stg	0.889	0.042	< 0.0001	(0.806–0.971)
Q106_stg	~~	Q106_stg	0.456	0.049	< 0.0001	(0.359–0.553)
Q107_stg	~~	Q107_stg	0.308	0.042	< 0.0001	(0.225–0.391)
Q108_stg	~~	Q108_stg	0.685	0.057	< 0.0001	(0.572–0.797)
Q109_stg	~~	Q109_stg	0.244	0.064	< 0.0001	(0.117–0.37)
Q204_stg	~~	Q204_stg	0.510	0.047	< 0.0001	(0.418–0.602)
Q205_stg	~~	Q205_stg	0.249	0.030	< 0.0001	(0.191–0.307)
Q206_stg	~~	Q206_stg	0.220	0.027	< 0.0001	(0.167–0.273)
Q207_stg	~~	Q207_stg	0.384	0.040	< 0.0001	(0.306–0.463)
Q208_stg	~~	Q208_stg	0.456	0.044	< 0.0001	(0.37–0.543)
Q209_stg	~~	Q209_stg	0.108	0.016	< 0.0001	(0.076–0.141)
Q210_stg	~~	Q210_stg	0.128	0.018	< 0.0001	(0.092–0.164)

Q211_stg	~~	Q211_stg	0.337	0.037	< 0.0001	(0.264–0.41)
Q212_stg	~~	Q212_stg	0.760	0.048	< 0.0001	(0.666–0.855)
Q203_stg	~~	Q203_stg	0.224	0.027	< 0.0001	(0.17–0.277)
Q213_stg	~~	Q213_stg	0.237	0.092	0.010	(0.056–0.418)
Q214_stg	~~	Q214_stg	0.471	0.075	< 0.0001	(0.324–0.618)
Q215_stg	~~	Q215_stg	0.721	0.058	< 0.0001	(0.608–0.835)
Q201_stg	~~	Q201_stg	0.172	0.036	< 0.0001	(0.102–0.242)
Q202_stg	~~	Q202_stg	0.003	0.036	0.928	(-0.066–0.073)
Avoidance	~~	Avoidance	0.423	0.071	< 0.0001	(0.283–0.563)
Personal_responsibility	~~	Personal_responsibility	0.543	0.074	< 0.0001	(0.398–0.689)
Severity	~~	Severity	0.254	0.082	0.002	(0.093–0.415)
Policy_opposition	~~	Policy_opposition	0.998	0.007	< 0.0001	(0.985–1.011)
Awkwardness	~~	Awkwardness	0.776	0.060	< 0.0001	(0.659–0.893)
EQ-5D-5L	~~	EQ-5D-5L	0.975	0.022	< 0.0001	(0.932–1.018)
K6	~~	K6	0.957	0.029	< 0.0001	(0.901–1.013)
EQ-5D-5L	~~	K6	-0.411	0.053	< 0.0001	(-0.514–-0.307)

* Operator "=~" indicates the left-hand side latent variable measured by the right-hand side variable, "~" indicates the left-hand side variable regressed on the right-hand side variable, and "~~" indicates the left-hand side variable correlated with the right-hand side variable.
